# Supplementary material for: Intersection of phosphate transport, oxidative stress and TOR signalling in Candida albicans virulence
Source: PLoS Pathog. 2018 Jul 30;14(7):e1007076. doi: 10.1371/journal.ppat.1007076 (PMC6085062; doi:10.1371/journal.ppat.1007076)

Supporting Information 1 Data

S1 Figure. Stress intensity-dependent haploinsufficiency of *pho84/pho84::PHO84* reintegrant cells. Overnight cultures were washed with normal saline and diluted to YPD with vehicle, 20 μM plumbagin, 22 μM plumbagin and 25 μM plumbagin to OD_{600}=0.1. OD_{600} was monitored every 15 minutes. Strains used were: +/+, JKC915; -/-, JKC1450 and -/-/+, JKC1588. 25 μM plumbagin depicts the same experiment as that shown in Fig. 3A. Representative of 3 biological replicates, error bars show SD of 3 technical replicates.

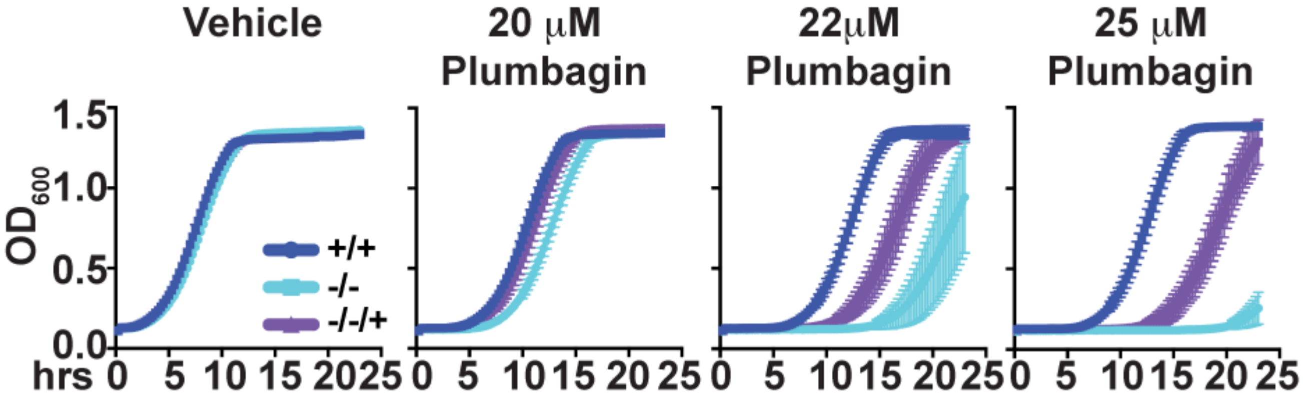

S2 Figure. Pho84 does not affect induction of phagocytosis or ROS production by neutrophils. (A) Percent survival of *C. albicans* cells after incubation of HL-60 derived neutrophils with +/+[1], SC5314; +/+[2], JKC915; -/-, JKC1450 and -/-/+, JKC1500 at a 20:1 phagocyte: fungus ratio. **p*<0.01; ns non-significant. (B) +/+ (*PHO84/PHO84* $_{promoter}$*ACT1-GFP*), JKC1648; -/- (*pho84/pho84* $_{promoter}$*ACT1-GFP*), JKC1651 and -/-/+ (*pho84/pho84::PHO84* $_{promoter}$*ACT1-GFP*), JKC1653 cells were incubated with neutrophils at M.O.I. 2 and M.O.I. 10. Phagocytosing neutrophils were quantified as CD11b+ GFP+ Cells. (C) Intracellular ROS production by neutrophils was measured after stimulation with *C. albicans* yeast at M.O.I. 2 for 30 minutes. (D) Extracellular ROS production was measured by incubation with *C. albicans* yeast at M.O.I. 2 for 1 hour in the presence of 100mM Cytochrome C. Strains used were: +/+, JKC915; -/-, JKC1450 and -/-/+, JKC1588. A shows representative of 3 biological replicates; B, C: representative of 2 biological replicates is shown; error bars SD of 3 technical replicates.

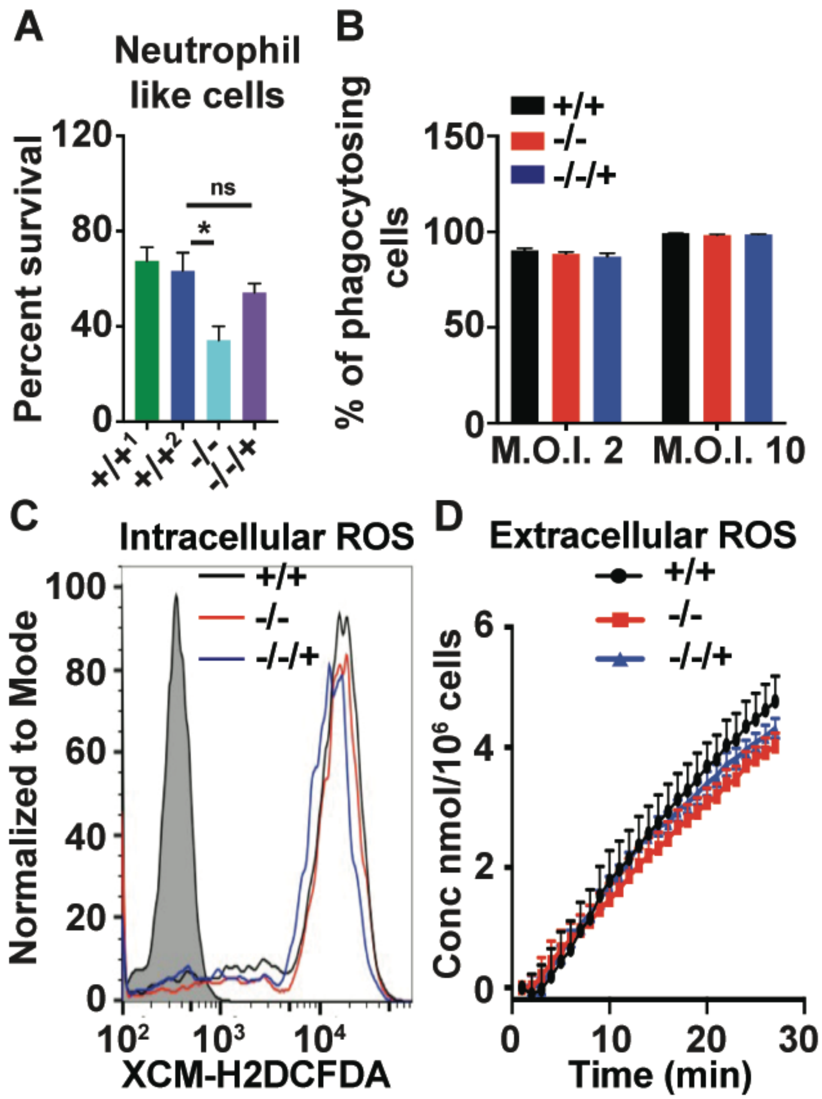

S3 Figure. In unstressed *pho84* cells, Hog1 phosphorylation is not measurably increased, while upon extrinsic peroxide exposure P-Hog1 increase is more intense and prolonged in *pho84* cells than in wild type. Western blot of cells incubated in YPD without H_2O_2 (0 minutes) and in YPD containing 5mM H_2O_2 for the stated times. The membrane was probed for phosphorylated Hog1 (P-Hog1), total Hog1, and loading control Histone H3. Dens: ratio of densitometry of P-Hog1 signal to H3 signal. Strains, +/+, JKC915; -/-, JKC1450. Representative of 3 biological replicates.

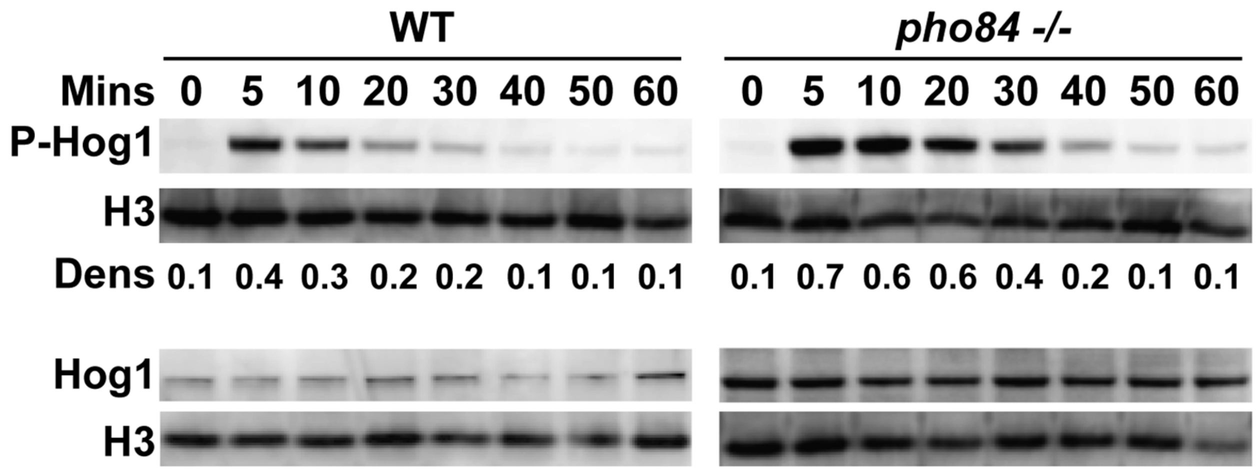

S4 Figure. Total intracellular metal SOD co-factors are not diminished in cells lacking *PHO84*. (A) Total intracellular copper of strains +/+, JKC915; -/-, JKC1450 and -/-/+, JKC1588, grown in normal SC medium with vehicle, 3 mM $MnCl_2$ and 3 mM $CuSO_4$ for 13 hours was measured by atomic absorption spectroscopy (AAS). (B) Total intracellular manganese of strains as in A, grown as in A, was measured by AAS. A and B show mean of 3 biological replicates obtained on different days; error bars SD of 3 biological replicates.

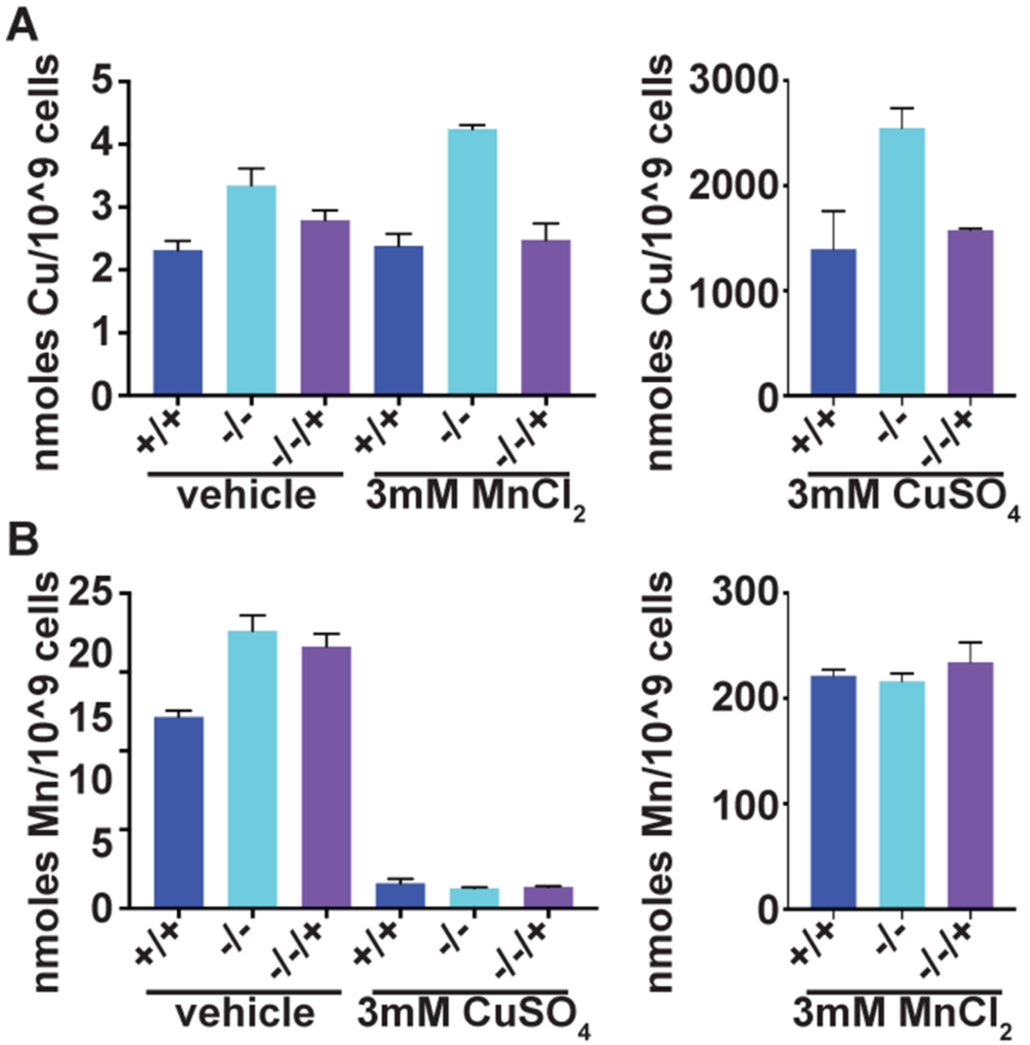

S5 Figure. *GTR1* overexpression does not suppress *pho84* mutant cells' hyphal morphogenesis defect, nor their hypersensitivity to killing by whole blood and neutrophils. (A) Hyphal growth of cells on RPMI1640 agar medium pH 7 at 37° C. Spot edges were imaged after 3 days to show length and density of hyphal filaments extending into the agar medium. Scale bar 1 mm. Strains: (1) *PHO84+/+* containing vector (JKC1594); (2) *PHO84*+/+ overexpressing *GTR1* (JKC1596); (3) *PHO84*+/+ overexpressing *GTR1*-GTP (JKC1619); (4) *pho84-/-* containing vector (JKC1598); (5) *pho84-/-* overexpressing *GTR1* (JKC1600); (6) *pho84-/-* overexpressing *GTR1*-GTP (JKC1616). (B) Percent survival of *C. albicans* cells after incubation with whole blood from healthy human volunteers. Strains as in A were inoculated into heparinized blood and plated onto agar medium at 0, 2 and 5 hours for calculation of colony forming units (cfu)/ml. (C) Percent survival of *C. albicans* cells after incubation with human neutrophils, at M.O.I. 2, calculated from cfu/ml at 0, 2 and 5 hours. Strains as in A. All representative of 3 biological replicates, error bars show SD of 3 technical replicates.

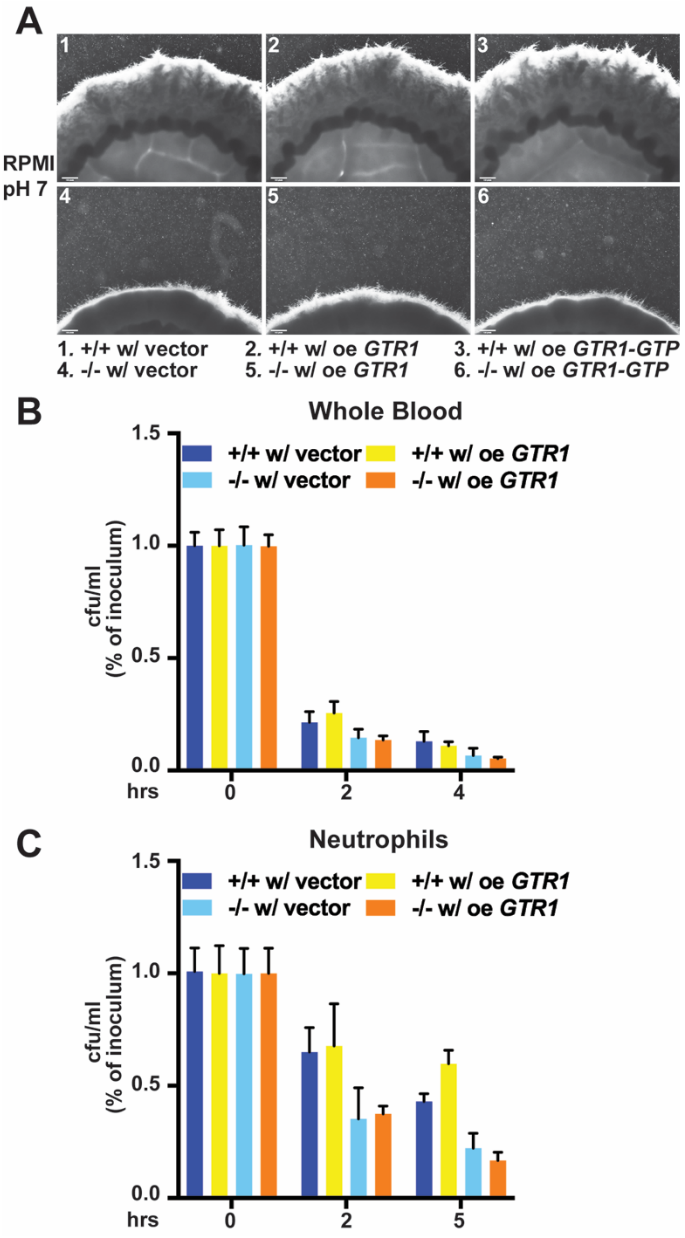

S6 Figure. *SOD3* overexpression does not suppress *pho84* mutant cells' hypersensitivity to killing by whole blood or neutrophils. (A) Percent survival of *C. albicans* cells after incubation with whole blood from healthy human volunteers. Strains: dark blue, *PHO84/PHO84*+/+ (JKC915); red, +/+ *tetO-SOD3/SOD3* (JKC1738); light blue, *pho84-/-* (JKC1450); green, -/- *tetO-SOD3/SOD3* (JKC1745). Cells were inoculated into heparinized blood and plated onto agar medium at 0, 2 and 5 hours for calculation of cfu/ml. (B) Percent survival of *C. albicans* cells after incubation with neutrophils at M.O.I. 2 for 0, 2 and 5 hours. Strains as in A. Representative of 3 biological replicates, error bars show SD of 3 technical replicates.

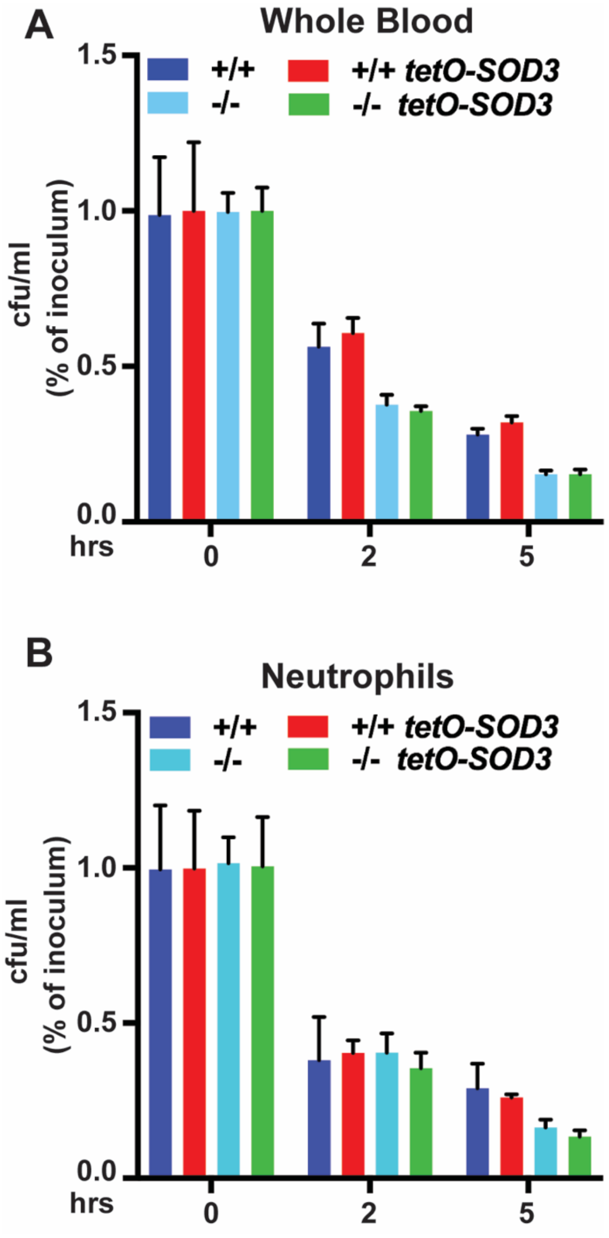

Supplement: S1 Data — (PDF) [file ppat.1007076.s001.pdf]
