# Supplementary material for: Intersection of phosphate transport, oxidative stress and TOR signalling in Candida albicans virulence
Source: PLoS Pathog. 2018 Jul 30;14(7):e1007076. doi: 10.1371/journal.ppat.1007076 (PMC6085062; doi:10.1371/journal.ppat.1007076)
Supplement: S1 Text — (PDF) [file ppat.1007076.s002.pdf]

Supporting Information 1 File

S1 Table. Strains used in this study

C. albicans strain name	Parent	Genotype	Strain background /construction	Reference
SC5314		Wild type	Bloodstream isolate	[1]
JKC915	SC5314	*HIS1/his1:: tetR-FRT*		[2]
JKC1450	JKC1423	*pho84::HIS1/pho84::ARG4*<br>*his1/his1:: tetR-FRT arg4/arg4*<br>*IRO1/iro1Δ::λimm*^{434} *URA3/ura3Δ::λimm*^{434}		[3]
JKC1583	JKC1423	*PHO84/pho84::HIS1*<br>*his1/his1:: tetR-FRT arg4/arg4::ARG4*<br>*IRO1/iro1Δ::λimm*^{434} *URA3/ura3Δ::λimm*^{434}		[3]
JKC1500	JKC1450	*PHO84-FRT/pho84::ARG4*<br>*his1/his1:: tetR-FRT arg4/arg4*<br>*URA3/ura3::λimm434 IRO1/iro1::λimm434*		[3]
JKC1588	JKC1500	*PHO84-FRT/pho84::ARG4 LEU2/leu2::C.d. HIS1*<br>*his1/his1:: tetR-FRT arg4/arg4*<br>*URA3/ura3::λimm434 IRO1/iro1Δ::λimm434*		[3]
JKC1594	JKC917	*ACT1/* $_{promoter}$*ACT1-NAT1-*$_{terminator}$*ACT1*<br>*his1/his1:: tetR-FRT arg4/arg4*<br>*IRO1/iro1Δ::λimm*^{434} *URA3/ura3Δ::λimm*^{434}		[3]
JKC1596	JKC917	*ACT1/* $_{promoter}$*ACT1-GTR1-NAT1-*$_{terminator}$*ACT1*<br>*his1/his1:: tetR-FRT arg4/arg4*<br>*IRO1/iro1Δ::λimm*^{434} *URA3/ura3Δ::λimm*^{434}		[3]
JKC1598	JKC1450	*ACT1/* $_{promoter}$*ACT1-NAT1-*$_{terminator}$*ACT1*<br>*pho84::HIS1/pho84::ARG4*<br>*his1/his1:: tetR-FRT arg4/arg4 IRO1/iro1Δ::λimm*^{434}<br>*URA3/ura3Δ::λimm*^{434}		[3]
JKC1600	JKC1450	*ACT1/* $_{promoter}$*ACT1-GTR1-NAT1-*$_{terminator}$*ACT1*<br>*pho84::HIS1/pho84::ARG4*<br>*his1/his1:: tetR-FRT arg4/arg4 IRO1/iro1Δ::λimm*^{434}<br>*URA3/ura3Δ::λimm*^{434}		[3]
JKC1616	JKC1450	*ACT1/* $_{promoter}$*ACT1-GTR1*^{Q67L}*-NAT1-*$_{terminator}$*ACT1*<br>*(GTR1-GTP) pho84::HIS1/pho84::ARG4*<br>*his1/his1:: tetR-FRT arg4/arg4 IRO1/iro1Δ::λimm*^{434}<br>*URA3/ura3Δ::λimm*^{434}		[3]
JKC1619	JKC917	*ACT1/* $_{promoter}$*ACT1-GTR1*^{Q67L}*-NAT1-*$_{terminator}$*ACT1*<br>*(GTR1-GTP) his1/his1:: tetR-FRT arg4/arg4*<br>*IRO1/iro1Δ::λimm*^{434} *URA3/ura3Δ::λimm*^{434}		[3]
JKC1648	JKC915	*ACT1/* $_{promoter}$*ACT1-GFP-NAT1-*$_{terminator}$*ACT1*<br>*his1/his1:: tetR-FRT*	JKC915 transformed with pJK1323, cut with BsrGI, to overexpress GFP	This work
JKC1651	JKC1450	*ACT1/* $_{promoter}$*ACT1-GFP-NAT1-*$_{terminator}$*ACT1*<br>*pho84::HIS1/pho84::ARG4*<br>*his1/his1:: tetR-FRT arg4/arg4 IRO1/iro1Δ::λimm*^{434}<br>*URA3/ura3Δ::λimm*^{434}	JKC1450 transformed with pJK1323, cut with BsrGI, to overexpress GFP	This work
JKC1653	JKC1588	*ACT1/* $_{promoter}$*ACT1-GFP-NAT1-*$_{terminator}$*ACT1*<br>*PHO84-FRT/pho84::ARG4 LEU2/leu2::C.d. HIS1*<br>*his1/his1:: tetR-FRT arg4/arg4*<br>*URA3/ura3::λimm434 IRO1/iro1Δ::λimm434*	JKC1588 transformed with pJK1323, cut with BsrGI, to overexpress GFP	This work
JKC1361	JKC917	*his1/his1:: tetR-FRT arg4/ARG4*<br>*IRO1/iro1Δ::λimm*^{434} *URA3/ura3Δ::λimm*^{434}		[3]
JKC1717	JKC915	*HIS1/his1::tetR-FRT SOD3/FLP-NAT-tetO-SOD3*	JKC915 transformed with pJK1351, cut with KpnI/NcoI, to control *SOD3* expression from *tetO*, isolate #3	This work
JKC1738	JKC1717	*HIS1/his1::tetR-FRT SOD3/FRT-tetO-SOD3*	JKC1717 after recombination of FRTs following FLP induction	This work
JKC1720	JKC915	*HIS1/his1::tetR-FRT SOD3/FLP-NAT-tetO-SOD3*	JKC915 transformed with pJK1351, cut with	This work

			KpnI/NcoI, to control *SOD3* expression from *tetO*, isolate #4	
JKC1742	JKC1720	*HIS1/his1::tetR-FRT SOD3/FRT-tetO-SOD3*	JKC1720 after recombination of FRTs following FLP induction	This work
JKC1726	JKC1450	*pho84::ARG4/pho84::HIS1 SOD3/FLP-NAT-tetO-SOD3*	JKC1450 transformed with pJK1351, cut with KpnI/NcoI, to control *SOD3* expression from *tetO*, isolate #5	This work
JKC1745	JKC1726	*pho84::ARG4/pho84::HIS1 SOD3/FRT-tetO-SOD3*	JKC1726 after recombination of FRTs following FLP induction	This work
JKC1729	JKC1450	*pho84::ARG4/pho84::HIS1 SOD3/FLP-NAT-tetO-SOD3*	JKC1450 transformed with pJK1351, cut with KpnI/NcoI, to control *SOD3* expression from *tetO*, isolate #7	This work
JKC1751	JKC1729	*pho84::ARG4/pho84::HIS1 SOD3/FRT-tetO-SOD3*	JKC1729 after recombination of FRTs following FLP induction	This work
JKC1769	JKC915	*HIS1/his1::tetR-FRT SOD3/FLP-NAT-$_{promoter}$MAL2-SOD3*	JKC915 transformed with pJK1353, cut with KpnI/NcoI, to control *SOD3* expression from $_{promoter}$*MAL2*, isolate #3	This work
JKC1776	JKC1769	*HIS1/his1::tetR-FRT SOD3/FRT-$_{promoter}$MAL2-SOD3*	JKC1769 after recombination of FRTs following FLP induction	This work
JKC1772	JKC1450	*pho84::ARG4/pho84::HIS1 SOD3/FLP-NAT-$_{promoter}$MAL2-SOD3*	JKC1450 transformed with pJK1353, cut with KpnI/NcoI, to control *SOD3* expression from $_{promoter}$*MAL2*, isolate #1	This work
JKC1780	JKC1772	*pho84::ARG4/pho84::HIS1 SOD3/FRT-$_{promoter}$MAL2-SOD3*	JKC1772 after recombination of FRTs following FLP induction	This work

S2 Table. Plasmids used in this study

**Plasmid**	**Description**	**Source (Reference)**
pGFP-HIS1		[4]
pJK1027	pAU34 with *URA3* disrupted by *Ag*$_{promoter}$*TEF1-NAT1-Ag*$_{terminator}$*TEF1*	[5]
pJK1085	*CaTPK1* ligated into XmaI sites of pJK1027, used as cloning vector to replace *TPK1* with GFP.	This work
pJK1323	*pACT1-GFP*, product of fjk1615/rjk1633 using *pGFP-HIS1* as template, ligated into XmaI/ClaI sites of pJK1085.	This work
pJK1000	*FLP-NAT1-tetO-PES1*, derived from Litmus 28 (NEB). Used as a template for sub-cloning *SOD3* homology sequences to generate *tetO-SOD3.*	[2]
pJK1351	*FLP-NAT1-tetO-SOD3*, derived from pJK1000. Product of fjk1821 and rjk1822 using SC5314 genomic DNA as template was ligated into SacII/NcoI sites of pJK1000; product of fjk1819 and rjk1820 using SC5314 genomic DNA as template was ligated into KpnI/ApaI sites of pJK1000.	This work
pJK896	*FLP-NAT1-pMAL2-PES1*	[2]
pJK1353	*FLP-NAT1-pMAL2-SOD3*, derived from pJK1351, *MAL2* promoter was excised from pJK896 using NotI/SacII, and ligated into p1351 that was digested at the same sites.	This work

S3 Table. Primers used in this study

**Primer name**	**Purpose**	**Sequence 5' to 3'**
fjk1615	Forward to amplify GFP using *pGFP-HIS1* as template	CCTGCTATCGATATGTCTAAAGGTGAAGAATTAT
rjk1633	Reverse to amplify GFP using *pGFP-HIS1* as template, eliminating BsrGI site	GCAGCTCCCGGGTTATTTGTATAATTCATCCATACCATGG
fjk1514	Forward to verify 5' end of *pACT1-GFP*	GACACCTAACTAATAAACC
rjk1515	Reverse to verify 5' end of *pACT1-GFP*	CCAGTAAATAATTCTTCACC

fjk1516	Forward to verify 3' end of *pACT1-GFP*	CAATCAATTAGAATTGAAGC
rjk1517	Reverse to verify 3' end of *pACT1-GFP*	GGAATTGTGAGCGGATAAC
fjk1819	Forward to amplify *SOD3* promoter 5’ homologous sequence, using SC5314 genomic DNA as template	CATCCGggtaccCGAAGCTACTAAACTGGTG
rjk1820	Reverse to amplify *SOD3* promoter 5’ homologous sequence, using SC5314 genomic DNA as template	GATCgggcccGCTTTTATACCCAGAGAGTGCTG
fjk1821	Forward to amplify *SOD3* open reading frame 3’ homologous sequence, using SC5314 genomic DNA as template	GGATCCccgcggATGATTACCGAAAACGAAAAG
rjk1822	Reverse to amplify *SOD3* 3’ homologous sequence, using SC5314 genomic DNA as template	CTCATGccatggGACCCATACTGAGCAACGAT
fjk1827	Forward to verify integration of *tetO-SOD3* and *pMAL2-SOD3* at 5’ end	GCCGACCAGCTCACCCTTAT
rjk1339	Reverse to verify integration of *tetO-SOD3* and *pMAL2-SOD3* at 5’ end	TGGTGTGTTGTTGACAGGCAAC
po547	Reverse from within *FLP-NAT1* cassette to verify integration of *SOD3* at 5’ end	CTAGAACTAGTGGATCCGAAG
fjk1835	Forward to verify integration of *tetO-SOD3* at 3’ end from within *OP4*	TGTCGTTTCTGATGGGCTTT
fjk741	Forward to verify integration of *pMAL2-SOD3* at 3’ end	GTCGCAgcggccgcGATTGATATTTTTGTCTAGTACC
rjk1828	Reverse to verify integration of *tetO-SOD3* and *pMAL2-SOD3* at 3’ end	CCTGATTGGCAGTAGTGACC
fjk1215	Forward to verify integration of *tetO-SOD3* at 3’ end from within *NAT1*	CCAACTACGTCTACTACTACG

S4 Table. Antibodies used in this study

Purpose	Antigen recognized	Species	Source or Reference
	Hog1	mouse	Santa Cruz Biotechnology, cat. # sc-165978
	Phosphorylated Hog1	rabbit	Cell Signaling Technology, cat. #4511
	Sod3	rabbit	Valeria C. Culotta
loading control	PSTAIRE	rabbit	Santa Cruz Biotechnology, cat. # sc-53
loading control	Tubulin	rat	Abcam, cat. # ab6161
loading control	Histone H3	rabbit	Cell Signaling Technology, cat. #4499
secondary	Mouse Ig		Santa Cruz Biotechnology, cat. #sc-516102
secondary	Rabbit Ig	bovine	Cell Signaling Technology, cat. # 7074s
secondary	Rat Ig	goat	Santa Cruz Biotechnology, cat. #97057
secondary	Goat Ig	donkey	Santa Cruz Biotechnology, cat. #sc-2020

Supporting Information References

1. Fonzi WA, Irwin MY. Isogenic strain construction and gene mapping in *Candida albicans*. Genetics. 1993;134(3):717-28.
2. Shen J, Cowen LE, Griffin AM, Chan L, Köhler JR. The *Candida albicans* pescadillo homolog is required for normal hypha-to-yeast morphogenesis and yeast proliferation. Proceedings of the National Academy of Sciences of the United States of America. 2008;105(52):20918-23.

3. Liu NN, Flanagan PR, Zeng J, Jani NM, Cardenas ME, Moran GP, et al. Phosphate is the third nutrient monitored by TOR in *Candida albicans* and provides a target for fungal-specific indirect TOR inhibition. Proceedings of the National Academy of Sciences of the United States of America. 2017. 2017 Jun 13;114(24):6346-6351.
4. Gerami-Nejad M, Berman J, Gale CA. Cassettes for PCR-mediated construction of green, yellow, and cyan fluorescent protein fusions in *Candida albicans*. Yeast. 2001;18(9):859-64.
5. Patenaude C, Zhang Y, Cormack B, Köhler J, Rao R. Essential role for vacuolar acidification in *Candida albicans* virulence. The Journal of Biological Chemistry. 2013;288(36):26256-64.
